# The Risk of Chronic Diseases in Individuals Responding to a Measure for the Initial Screening of Depression and Reported Feelings of Being Down, Depressed, or Hopeless

**DOI:** 10.7759/cureus.17634

**Published:** 2021-09-01

**Authors:** Mohammed Khatib, Nathan Badillo, Payal Kahar, Deepesh Khanna

**Affiliations:** 1 Dr. Kiran C. Patel College of Osteopathic Medicine, Nova Southeastern University, Clearwater, USA; 2 Department of Health Sciences, Florida Gulf Coast University, Fort Myers, USA

**Keywords:** chronic disease, depression, preventive medicine, mental health, behavioral health, patient health questionnaire-2, hypertension, dyslipidemia, asthma, diabetes

## Abstract

Introduction

Feeling down, depressed, or hopeless may provide a comprehensive measure for physicians to utilize, allowing a possible way to assess risk for chronic diseases.

Methods

A face-to-face, in-home, validated survey was conducted on participants aged 16 and older. Trained interviewers administered the questionnaire through the Computer-Assisted Personal Interview (CAPI) system. Through this measure, responses such as feelings of depression, diagnosis of high cholesterol, high blood pressure, diabetes, asthma, being overweight, coronary heart disease, and cancer or malignancy were recorded. Statistical analysis was conducted by descriptive analysis, Chi-Square test, and multinomial regression analysis. *Results*: Data are presented as a mean ± SD and percentage. A total of 10560 individuals participated in the survey. Of participants reporting feeling down, depressed, or hopeless almost every day, 54.3% reported high blood pressure (χ2=116.108, *p*= 0.000), 44.1% with high cholesterol level (χ2=54.89, *p*= 0.000), 22.9% with diabetes (χ^2^=91.09, *p*= 0.000), 25.0% with asthma (χ2=93.83, *p*= 0.000), 49.5% had a doctor tell them they were overweight (χ2=59.32, *p*= 0.000), 8.2% had coronary heart disease (χ2=32.39, *p*= 0.000), and 11.4% that had cancer or malignancy (χ2=7.73, *p*= 0.655). This is compared to individuals who reported no feelings of depression, with 34.2% having high blood pressure, 32.2% with high cholesterol, 12.9% with diabetes, 14.1% told they had asthma, 14.1% told they were overweight, 3.9% with coronary heart disease, and 9.4% who had cancer or malignancy.

Conclusion

The assessment of feeling down, depressed, or hopeless is significantly associated with the risk of certain chronic diseases, with those who reported feelings of depression nearly every day at the highest risk.

## Introduction

Chronic diseases are currently a nationwide issue comprising 75% of total healthcare costs as well as most deaths within the US, indicating a need for restructuring of health care delivery [1]. Hypertension, respiratory diseases, chronic mental conditions, obesity, and arthritis are among the chronic diseases with the highest prevalence [2,3]. A large percentage of these chronic diseases are comorbid, with increased incidence in elderly populations [4]. The association between health and depression has been a long-established trend in medicine. Studies have found correlations between depression and multiple chronic diseases; hypertension [5], obesity [6], asthma [7], diabetes [8], and dyslipidemia [9]. These studies indicate the importance of early diagnosis of depression in at-risk populations and the benefit of implementing behavioral and preventive treatment, especially considering the comorbid nature of chronic diseases and their increased incidence with age. Consideration for the overwhelming nature of chronic diseases must be considered to understand the full impact a patient undergoes with such a diagnosis. A literature review of emotion regulation studies found a potential link between greater difficulties with emotion regulation and the presence of chronic disease, as well as poorer physical function [10]. These patients are burdened with physiological and psychological challenges, causing emotional reserves to be depleted, and with that, self-care management falls, potentiating the severity of the disease. A study highlighted the need to identify at-risk individuals based on mental health and demographic factors [10]. The results of the study showed that there is a strong correlation between more adaptive emotion regulation with increased age, decreased stress, male gender, and higher education. With evidence suggesting the application of emotion-regulation interventions to improve health and wellbeing in at-risk and clinical populations [11], the clinical importance in early detection of depression in individuals diagnosed with chronic disease is highlighted. The American College of Preventive Medicine recommends that all primary care practices screen and have systems to ensure accurate diagnosis and treatment of depression for adults [12]. Addressing behavioral health within the clinic allows significantly improved control of medical disease and depression with reduced cost [13]. There is strong evidence for retooling primary care practice with collaboration from psychologists to address these issues between behavior and chronic illness [14].

As retooling and restructuring is a time and resources heavy process, clinicians' application of screening tools can bridge the health care delivery gap. For screenings of major depression, the Patient Health Questionnaire-2 (PHQ-2) is utilized for initial screening in the clinical setting [15]. It is adapted from the Patient Health Questionnaire-9 (PHQ-9)'s first two questions that are used for further evaluation. Both measures are components of the Patient Health Questionnaire, incorporating Diagnostic and Statistical Manual of Mental Disorders, fourth edition (DSM-IV) depression criteria for the screening and diagnosis, as well as monitoring of treatments [16]. A meta-analysis of the PHQ-2 found the measure to have significantly greater specificity over the PHQ-9 linear cut-off method. Its use as an initial screening allows less burden on clinicians with its ease of use, sensitivity, and specificity, especially within the primary care setting [17,18].

Describing the accuracy of the measures is warranted to provide further validity and evidence of both the correlation between depression and chronic diseases and support for the integration of short questionnaires within the primary care setting. The current study examined the prevalence of chronic diseases among those who self-reported feeling down, depressed, or hopeless using the PHQ-2 questions.

## Materials and methods

Study Population

The Centers for Disease Control and Prevention (CDC) performed the National Health and Nutrition Examination Survey (NHANES), a face-to-face, in-home, validated survey conducted on participants aged 16 and older. Trained interviewers administered the questionnaire through the Computer-Assisted Personal Interview (CAPI) system. Through this, measures such as feelings of depression, asthma, high cholesterol, high blood pressure, diabetes, and obesity were recorded [19].

Between 2013 and 2016, a total of 29,659 were selected for NHANES, and of this 20,146 completed the interview with 19,357 being examined. A total of 19741 participants completed both the mental health sections and physical health sections of the interview. After the exclusion of respondents with missing data (9181), the final working sample size was 10560 Respondents, 5378 women, and 5182 men. The average age of men and women was 31.1 and 32.2 years respectively [19].

Measures

To measure the severity of depression, respondent answers were used for the following mental health depression screener asked by NHANES, variable DPQ020: "Over the last two weeks, how often have you been bothered by any of the following problems? Feeling down, depressed, or hopeless." This question comprised one of the two items for the PHQ-2. A validated measure with good sensitivity and specificity for detecting major depression [18].

With respect to our primary outcome measure of chronic disease, our response variables of interest were high blood pressure, diabetes, asthma, overweight, coronary heart disease, and cancer or malignancy. The respondent answers for the following variables of chronic disease followed a similar survey question, ever told you had/ Doctor ever said you had any of the above-mentioned chronic diseases.

The results of the statistical analysis were stratified as gender to understand how responses to the health depression screener affect the risk of chronic disease vary between men and women.

Statistical Analysis

Individual responses to the measure of depression were compared to the incidence of chronic medical issues by cross-tabulation and chi-squared tests. Cross tabulations were stratified by gender, allowing to examine how depression affects the incidence of chronic diseases in men and women. All data are reported as percentages and a total number of participants in each group. Analyses were performed using IBM SPSS version 26 (IBM Inc., Armonk, New York) [20].

## Results

Results

Groups were created depending on answers to the days felt feeling down, depressed, or hopeless (see Table 1). Statistical analysis showed a stepwise progression in the percentage of groups suffering from chronic disease, with those who reported feelings of depression nearly every day reported higher prevalence for each chronic disease.

**Table 1:** Sample characteristics

	Men (n=5182)	Women (n=5378)	Total (n=10560)
Feeling down, depressed, or hopeless	%	%	%
None of the Days	75.3	76.0	75.7
Several days	16.7	16.9	16.8
More than half the days	4.3	3.8	4.0
Nearly Every Day	3.6	3.2	3.4

**Figure 1:**
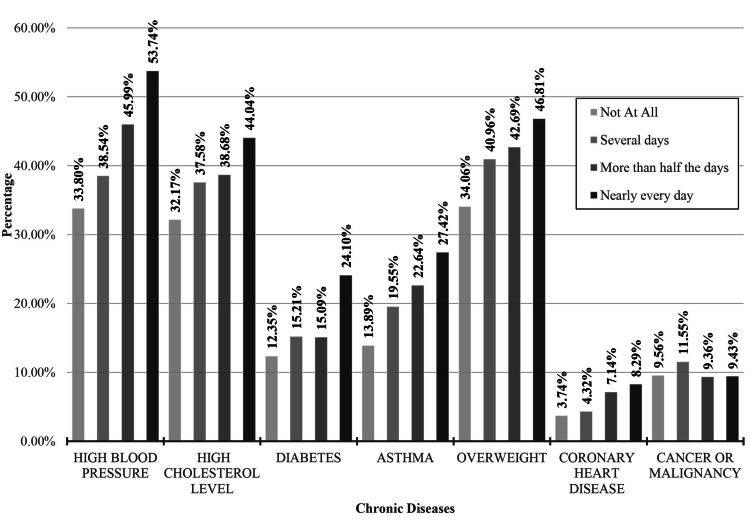
Comparison of reported high blood pressure, cholesterol, diabetes, asthma, being overweight, coronary heart disease, and cancer or malignancy within self-reported feelings of depression

A total of six chronic diseases were significantly associated with the odds of respondents' answers to the measure of depression (Table 2): high blood pressure, high cholesterol levels, diabetes, asthma, overweight, and heart disease. The participants that have reported feeling depressed, down, or hopeless nearly every day reported the highest percentages for high blood pressure at 53.7% (χ2=116.104, p=0.000), 44.0% with high cholesterol levels (χ2=54.893156, p=0.000), 24.1% reporting diabetes (χ2=54.893, p=0.000), 27.4% with asthma (χ2=93.836, p= 0.000), 46.8% being overweight (χ2=59.319, p=0.000), and 8.3% with coronary heart disease (χ2=32.390, p=0.000) with the exception of cancer at 9.4% of the group (χ2=7.732, p=0 .655). The participants who reported no feelings of being down, depressed, or hopeless had the lowest incidence of chronic disease with 33.8% having high blood pressure, 32.1% with high cholesterol levels, 12.4% with diabetes, 34.1% with asthma, 3.7% overweight, and 9.6% with coronary heart disease. Comparison of reported high blood pressure, cholesterol, diabetes, asthma, being overweight, coronary heart disease, and cancer or malignancy within self-reported feelings of depression is presented in Figure 1.

**Table 2:** Comparison of reported high blood pressure, cholesterol, diabetes, asthma within self-reported feelings of depression * p<0.05

	Frequency of feeling down, depressed, or hopeless						
	Not at All		Several days			More than half the days	Nearly every day
Total	%		%			%	%
High blood pressure (n=2698) *	33.8		38.5			46	53.7
High cholesterol level (n=3564)	32.2		37.6			38.7	44
Doctor told you have diabetes (n=1408) *	12.4		15.2			15.1	24.1
Told you have asthma (n=1653) *	13.9		19.5			22.6	27.4
Doctor ever said you were overweight (n=3802) *	34.1		41			42.7	46.8
Ever told you had coronary heart disease (n=413) *	3.7		4.3			7.1	8.3
Ever told you had cancer or malignancy (n=988)	9.6		11.5			9.4	9.4

Table 3 displays a cross-tabulation between populations separated by their answer to the specific measure of depression with the percentage afflicted by response variables for chronic disease, stratified by gender. Between genders, the groups with the highest incidence of chronic disease were the respondents with feelings of depression nearly every day.

**Table 3:** Comparison of reported high blood pressure, cholesterol, diabetes, asthma within self-reported feelings of depression * p<0.05

	Frequency of feeling down, depressed, or hopeless			
	Not at All	Several days	More than half the days	Nearly every day
Men	%	%	%	%
High blood pressure (n=1843) *	34.2 (n=1334)	34.9 (n=303)	46.8 (n=104)	54.3 (n=102)
High cholesterol level (n=1728) *	32.2 (n= 1258)	34.3 (n=297)	40.2 (n=89)	44.1 (n=83)
Doctor told you have Diabetes (n=698) *	12.9 (n=503)	13.6 (n=118)	15.3 (n=34)	22.9 (n=43)
Told you have Asthma (n=819) *	14.1 (n=551)	20 (n=173)	21.6 (n=48)	25.0 (n=47)
Doctor ever said you were overweight (n=1891) *	34.6 (n=1349)	40.1 (n=348)	45.5 (n=101)	49.5 (n=93)
Ever told you had coronary heart disease (n=203)	3.9 (n=142)	3.9 (n=32)	6.6 (n=14)	8.2( n=15)
Ever told you had cancer or malignancy (n=471)	9.4 (n=344)	10.3 (n=84)	9.9 (n=21)	11.4 (n=21)
Women	%	%	%	%
High blood pressure (n=1931) *	33.4 (n=1364)	42.0 (n=381)	45 (n=91)	53.2 (n=92)
High cholesterol level (n=1836) *	32.1 (n=1313)	40.7 (n=370)	37.1 (n=75)	43.9 (n=76)
Doctor told you have Diabetes (n=710) *	11.8 (n=484)	16.7 (n=152)	14.9 (n=30)	25.4 (n=44)
Told you have Asthma (n=834) *	13.7 (n=559)	19.2 (n=174)	23.8 (n=48)	30.1 (n=52)
Doctor ever said you were overweight (n=1911) *	33.6 (n=1373)	41.7 (n=379)	39.6 (n=80)	43.9 (n=76)
Ever told you had coronary heart disease (n=210) *	3.6 (n=140)	4.7 (n=41)	7.7 (n=15)	8.4 (n=14)
Ever told you had cancer or malignancy (n=517)	9.7 (n=377)	12.7 (n=111)	8.8 (n=17)	7.2 (n=12)

However, within this group, more women reported incidence of being told they have diabetes (25.4%, χ2=43.694, p=0.000), asthma (30.1%, χ2=59.204, p=0.000), and coronary heart disease (8.4%, χ2=24.939, p=0.005) compared to men at 22.9%, 25.9, and 9.2% respectively. Women had the greatest change between groups when considering asthma, with a 16.4% change, as well as diabetes with a 13.6% change.

On the other hand, men reported a higher incidence of high blood pressure, high cholesterol levels, and being overweight. Men had the largest changes between groups when considering being overweight, with a 14.9% change, and high blood pressure, with a 20.1% change.

## Discussion

The question measured within this study is used within the PHQ-2 and PHQ-9 to screen for depression. A score > trhree out of six within the PHQ-2 indicates a likely incidence of a major depressive order. Those answering that they have felt down, depressed, or hopeless nearly every day have reached the optimal cutoff point of three for screening purposes, indicating a 38.4 positive predictive value, with 80% specificity and 82.8% sensitivity, disregarding their answer to the second question within the PHQ-2 [15]. There are indications for a PHQ-2 cut-point at >2 [15], which supports the findings in the use of psychological treatment to prevent major depression in people with subthreshold depression [15]. Statistical analysis of NHANES population sample data showed that participant answers to the measure "Over the last two weeks, how often have you been bothered by any of the following problems? Feeling down, depressed, or hopeless" were significantly associated with increased risk to several chronic diseases. Specifically, those who have reported feelings of depression nearly every day were at higher risks of hypertension, high cholesterol levels, diabetes, asthma, obesity, and coronary heart disease.

Application of these results should be applied as further validation of the PHQ-2 and PHQ-9 questionnaires, adding to the body of literature describing the questionnaires' efficacy in its clinical use, especially within primary care [16]. The PHQ-2 and PHQ-9 exhibited high sensitivity and specificity for screening major depression, indicating its beneficial use within the clinic [16]. There is an added potential use for these questionnaires as the question examined in this study has shown a direct association with the incidence of chronic disease in a stepwise fashion. This association can help to guide clinicians with screened individuals towards a comprehensive management plan targeting behavioral and physical health and hopefully encourage a more holistic view of the patient, underlining the importance of mental health and encouraging new modes of comprehensive care.

There is an increasing importance of addressing mental health and preventive medicine within the clinic, especially within populations suffering from chronic disease. The use of this measure can hopefully assist in obtaining a comprehensive view of the patient's mental and physical health. Greater importance should be placed on incorporating depression within chronic disease management considering the comorbid nature of the chronic disease and the emotional and physical toll it takes, stressing the importance of earlier consideration of behavioral and preventive treatment [15,19]. The primary care setting can benefit most from this, as it is better suited for managing both depression and chronic disease [15], and there is a noted discrepancy between the application of preventive service recommendations, due primarily to time constraints [15,19]. Supporting literature has stated a need for an adaption to new models of healthcare incorporating behavioral and chronic disease management [17,18], especially within the elderly where the high prevalence of late-life depression (LLD) increases the risk of morbidity and mortality [15]. While models of health care that incorporate behavioral health are seen to improve patient outcomes, large resource and time demands may counteract such programs' advantages [15] There is support in the use of the PHQ-2 and PHQ-9 within new models of primary care due to its ease of use, sensitivity, specificity, construct, and criterion validity in the screening of depression [15,21]. Statistical analysis of the question addressed within this study may provide further assistance for public health and clinical workers in obtaining an overview of patients' mental and physical health status. Due to the positive correlation between the number of days respondents felt down, depressed, or hopeless and chronic disease incidence, implementation of these questionaries and comprehensive analysis of patients may help bridge the gap in primary care practice, allowing adjustments to new models of healthcare services.

The findings of this study support the body of literature connecting depression to chronic disease. This is the first study of its kind that showed the correlation between the second question of the PHQ-2 questionnaire and the chronic diseases. Although this study determined a direct correlation, no inference can be drawn about the cause-and-effect relationship between depression and chronic disease. Studies are examining the possible interplay between patients with depression and chronic diseases proposing indications that it plays an important role in disease pathology [6, 8,9]. A review of emotion regulation studies found that overwhelming emotional demands depleted the reserves needed for day-to-day self-care management of chronic diseases, with the depletion of mental resources potentiating the severity of the disease [10]. It is important to know that a large percentage of these chronic diseases are comorbid and increase in incidence in the elderly [15].

Study Limitations

Although this study’s statistical analysis established a relationship between the measure of depression and the incidence of chronic disease, the cause of this relationship is unknown. Explanations such as the emotional resource models can attempt to describe the causality of mental illness and chronic disease progression [10]. Still, factors can be due to numerous extraneous confounding variables such as demographics. Incorporating demographic factors is necessary considering the increased incidence of chronic disease within elderly populations [15]. Incidence of depression and anxiety can differ due to race and education status, marital status, and financial level [15]. Further analysis incorporating NHANES demographic data is needed to see possible relations. This study assessed the efficacy of one measure of the PHQ-2. Additional statistical analysis is needed to evaluate the efficacy of the whole measure of the PHQ-2 and PHQ-9 with its correlation to chronic depression and the diagnosis of depression itself.

Due to the structure of the NHANES questionnaire data, chronic disease severity and duration are unknown. In the clinical setting, assessing the severity of the disease is an important step in evaluating the patient, especially for the incorporation of treatment regimens. This information could provide a multidimensional look at the connection between the extent of how health and depression are affected. Due to the self-report structure of NHANES, this study examines respondents’ subjective views regarding their health and emotion. There can exist variability in how a patient considers their mood and feelings, or if they consider their health issues to be a disease, such as a patient who was told they are pre-hypertensive by a health professional understanding it as having hypertension and reporting as such. This study consisted of a cross-sectional analysis due to the structure of the NHANES questionnaire data. Assessment of new disease diagnosis and feelings of depression could not be assessed over time. A longitudinal study assessing both chronic disease and depression incidence can further illustrate these two variables' relationship.

## Conclusions

This study results showed the merit of incorporating complete mental health assessment in the primary care practice as a way to fully evaluate whether the co-morbid diseases are being adequately treated and if so, maybe that would improve the behavioral aspect? The emotional demand and comorbid nature of chronic diseases can help to explain the relationship seen within the measure's direct relationship with chronic disease. However, further study is required to understand the relationship between the severity of depression to the severity or comorbidity of chronic illness seen in these populations. The results of the measure can serve as a guide but not final recommendations about how to incorporate behavioral and physical health.
